# Rare Concomitant Cesarean Scar Ectopic Pregnancy With Tubal Ectopic Pregnancy: A Case Report

**DOI:** 10.7759/cureus.37434

**Published:** 2023-04-11

**Authors:** Bharati Thakur, Tomesh Shrimali

**Affiliations:** 1 Pathology, Jawaharlal Nehru Medical College (JNMC) Datta Meghe Institute of Higher Education and Research, Wardha, IND; 2 Obstetrics and Gynecology, District Hospital Dhamtari, Dhamtari, IND

**Keywords:** cesarean, tubal, scar, pregnancy, ectopic

## Abstract

A cesarean ectopic pregnancy is the rarest of all pregnancies and occurs when pregnancy implants on a cesarean scar. Incidence estimated in overall cesarean delivery is 1/1,800-1/2,500. Following a cesarean procedure, this abnormal embryo implantation into the uterine myometrium and fibrous tissues has a high rate of morbidity and mortality. The most common type of ectopic pregnancy is tubal ectopic pregnancy, and both their incidence and their frequency are rising. Early detection and treatment of ectopic pregnancy are crucial since delays in these processes might result in maternal death and morbidity. We are reporting a case of two concurrent pregnancies in a 27-year-old female with two separate implantation sites. The simultaneous occurrence of a tubal and an ectopic scar pregnancy was highly unusual. Early detection and treatment of ectopic pregnancy help prevent complications, death, and morbidity because it is a potentially fatal condition.

## Introduction

An ectopic pregnancy occurs when a fertilized ovum implants outside the uterine cavity. One to two percent of pregnancies result in ectopic pregnancies, which can be fatal. The fallopian tube is the most standard site for ectopic implantation, accounting for 10% of all ectopic pregnancies. Additional places include the abdominal cavity, the cervix, under a cesarean scar, the ovary, the interstitial section of the fallopian tube, the myometrium, and others [1]. The classical triad of ectopic pregnancy includes amenorrhea, followed by abdominal pain and vaginal bleeding [2]. Ectopic pregnancy risk factors include previous extrauterine pregnancies, increasing maternal age, use of assisted reproductive technology, implantation of intra-uterine devices, use of progesterone-only pills, history of tubal ligation, ongoing sexually transmitted infection, and rising cesarean delivery rates [2]. The number of cases recorded has been trending upward, presumably due to the expanding use of cesarean sections [1]. Early identification could help prevent issues such as heavy bleeding and rupture of scar, that might need a hysterectomy. These could endanger the woman's life and affect her ability to conceive in the future [3]. Patients with stable vital signs can choose from a wider range of therapies, including conservative management. Therefore, radiologists and obstetricians/gynecologists must watch for this possibly fatal complication [4].

## Case presentation

**Figure 1:**
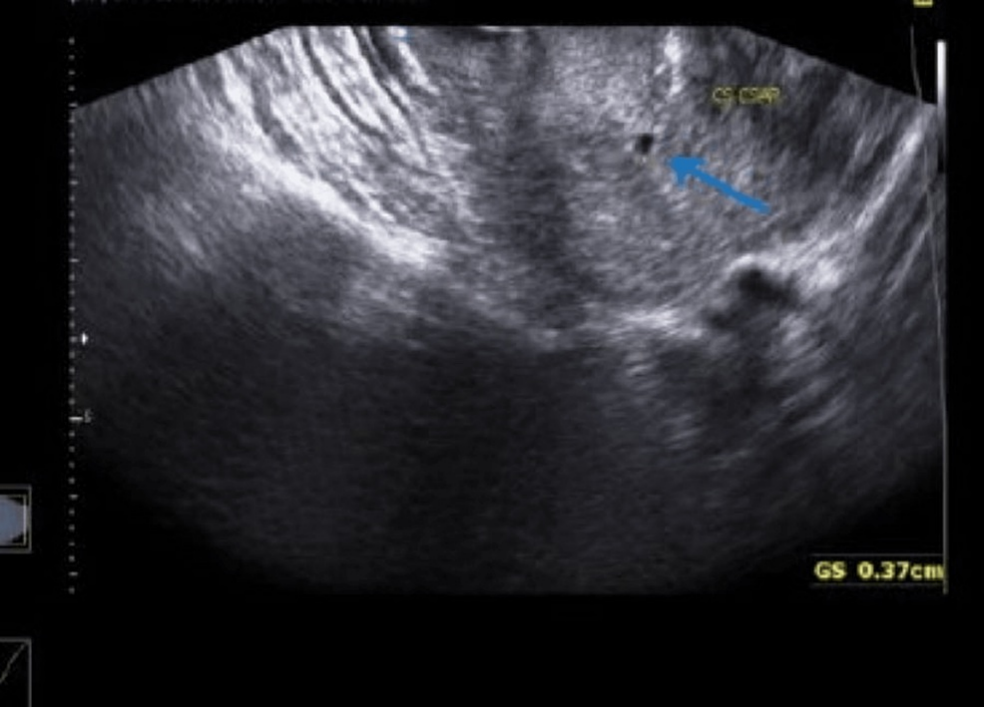
Scan showing very early intra-uterine gestational sac like structure noted at lower uterine segment, adjacent to cesarean section.

**Figure 2:**
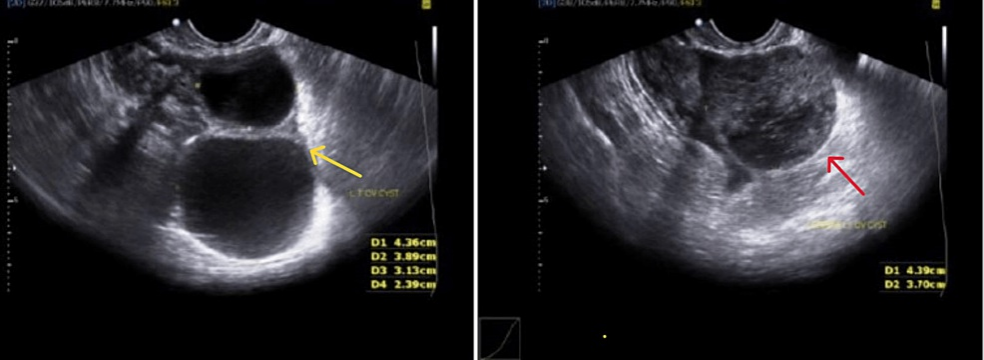
In the scan, yellow arrow is showing simple left ovarian cyst and red arrow is showing complex left ovarian cyst.

**Figure 3:**
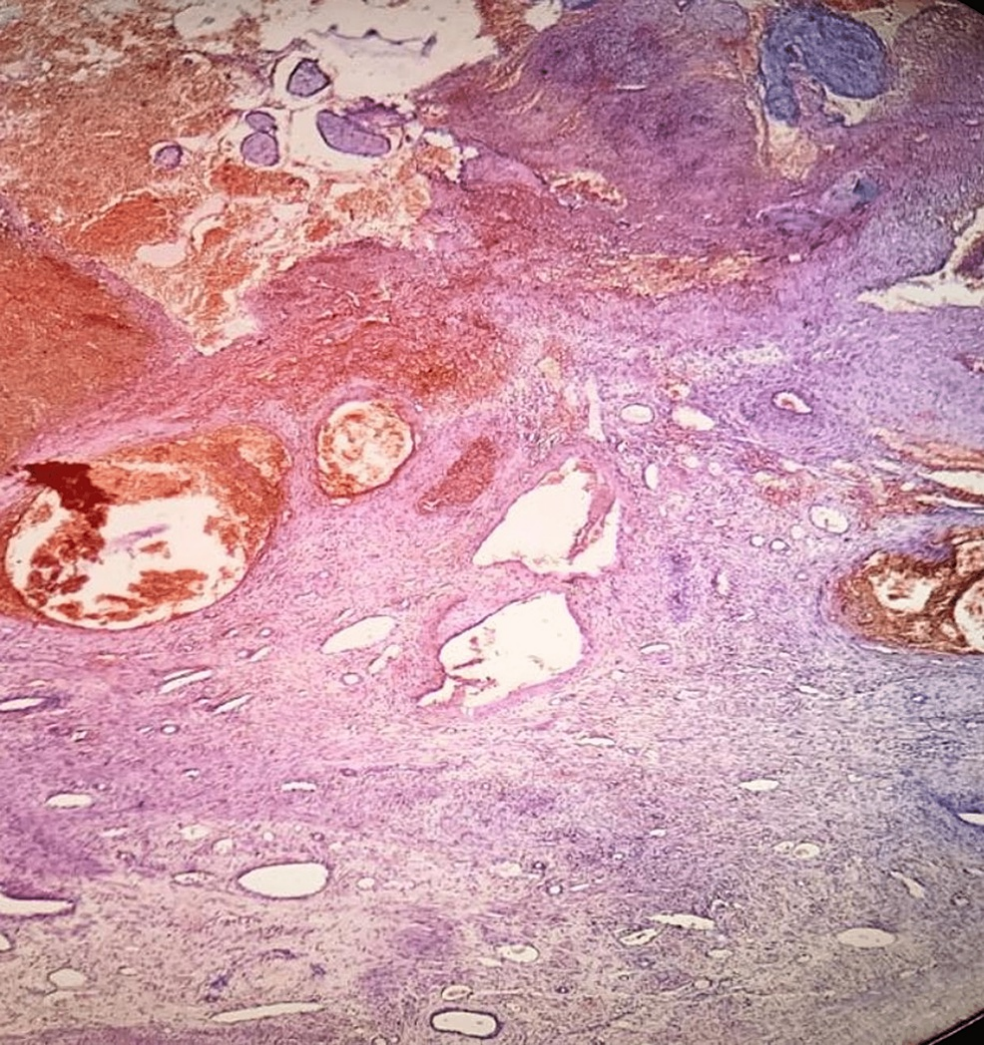
Villi and sac in a cesarean section scar surrounded with areas of hemorrhage and the fibrous tissue of scar (H&E, x10).

A 27-year-old woman with G2P1L1 presented to the Department of Obstetrics and Gynecology with 1.5 months of amenorrhea and complained of vaginal bleeding lasting two days. Her first birth took place by cesarean section two years ago. The patient was hospitalized and advised on Pelviabdominal ultrasonography (U.S.G), which suggested a very early cesarean scar ectopic pregnancy (Figure 1) with a mean gestational and sac diameter of 3.7 mm and complex and simple left ovarian cysts (Figure 2) with minimum fluid in the Pouch of Douglas. Her urine pregnancy test (UPT) also came up positive and her beta-human chorionic gonadotropin (bHCG) levels in pregnancy were 1880 mIU/ml. Her blood pressure was 108/66 mmHg and her pulse rate was 94/minute. Other standard investigations also came out within the expected range. Her abdomen was soft and non-tender, with a previous clean cesarean scar. The uterus was six weeks old upon abdominal palpation. Under spinal anesthesia, dilatation and curettage were carried out. A surgically removed product of conception (POC) was sent for a histopathology study. Histology confirmed the presence of decidua and chorionic villi in a cesarean section scar surrounded with fibrous tissue of scar and hemorrhages (Figure 3). After postoperative day 3, the patient's condition improved, she was discharged and advised to follow up after one week.

**Figure 4:**
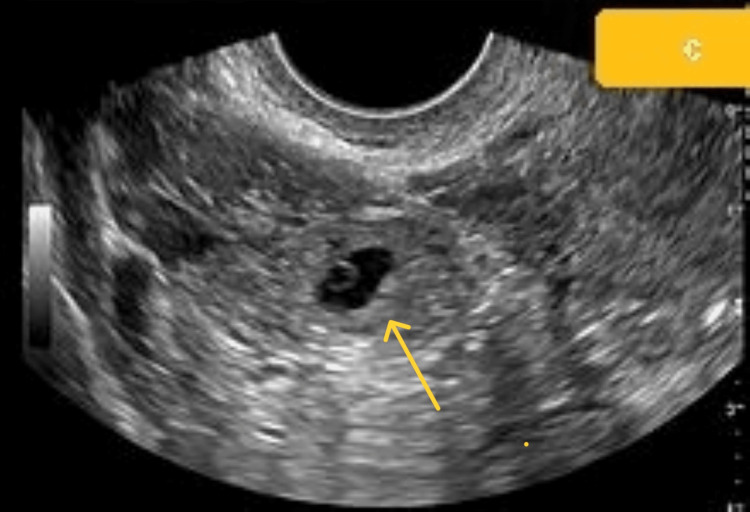
Scan showing left tubal ectopic pregnancy.

**Figure 5:**
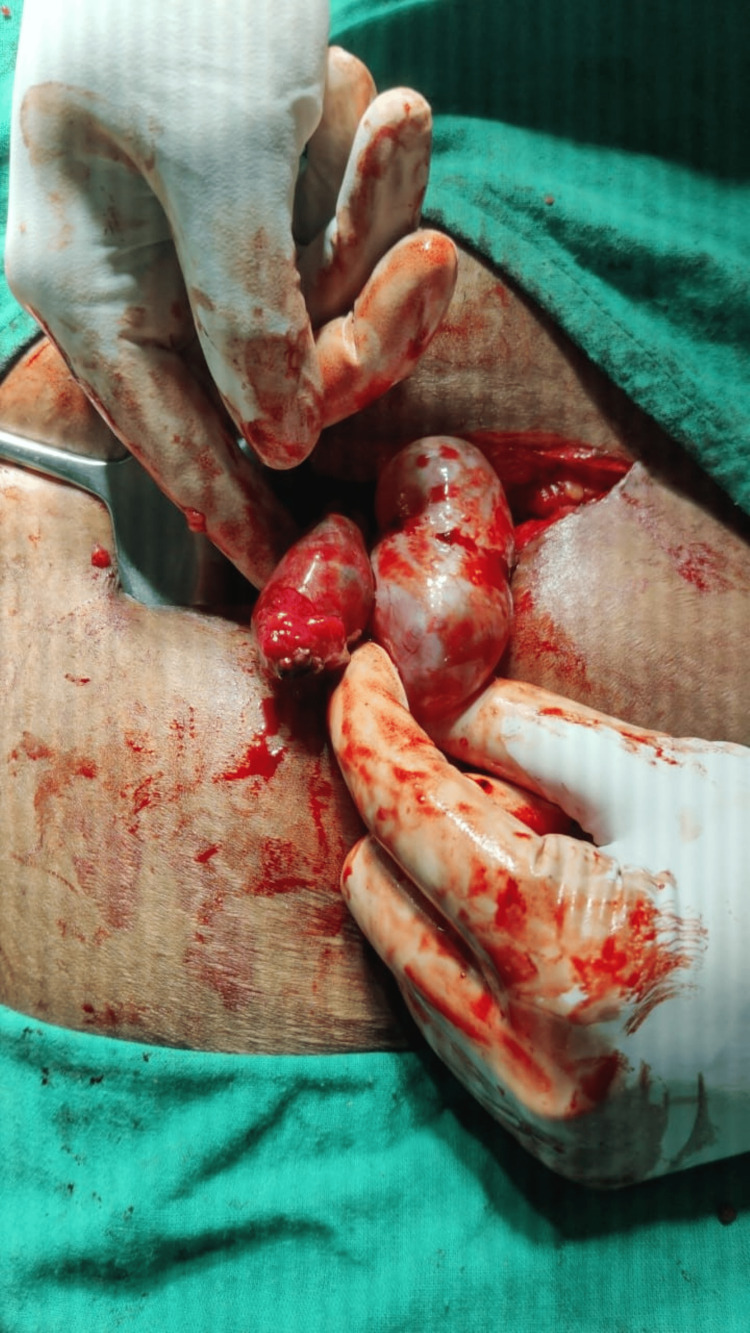
Scan showing intraoperative tubal ectopic pregnancy.

After a week, the patient returned to the emergency department with complaints of acute abdominal pain. Her blood pressure was 106/64 mmHg and pulse rate was 96/minute. She still had a positive UPT. The rest of the investigation was normal. Following conservative patient management, a U.S.G. Abdo-pelvis was performed. The results of the U.S.G. point to the likelihood of a left ectopic pregnancy due to an ill-defined heterogeneously hyperechoic complex left adnexal lesion with internal vascularity (left adnexal complex mass lesion) (Figure 4). During the exploratory laparotomy, a left complicated cyst with a ruptured left tubal ectopic pregnancy was discovered (Figure 5). Salpingectomy was then performed and sent for histological analysis.

**Figure 6:**
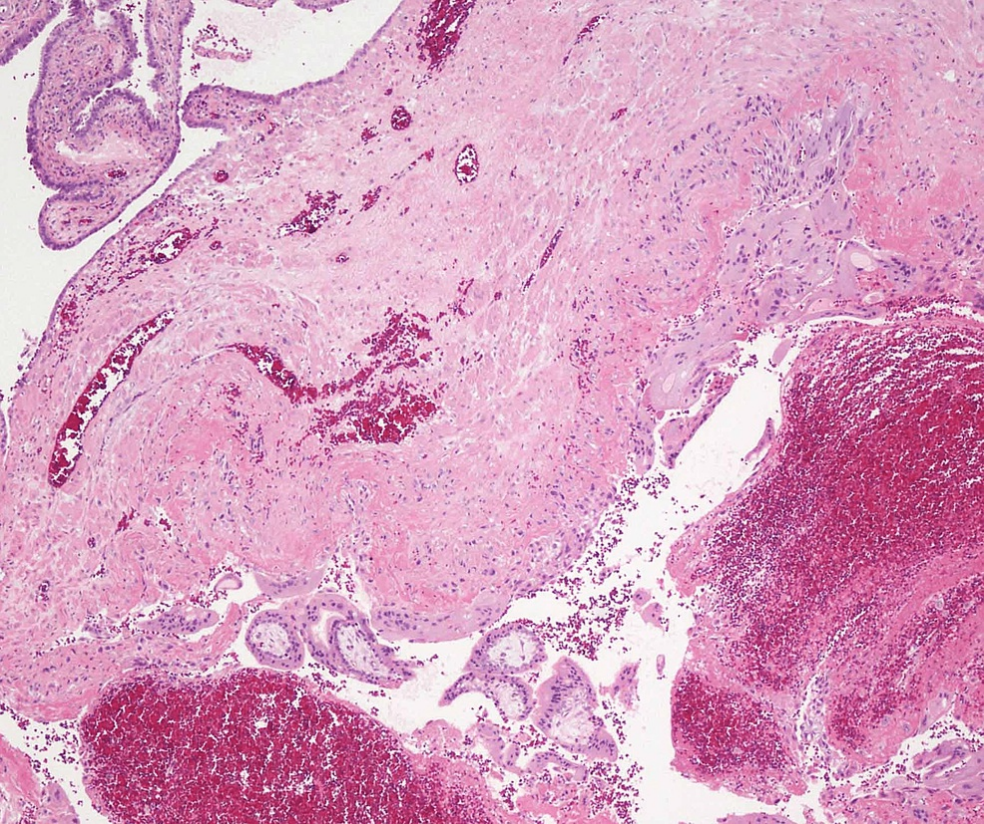
H&E 10x showing the product of conception with chorionic villi.

The grossly resected specimen consists of a 4.5 x 2.2 x 2.2 cm fallopian tube. The external surface is grayish black to brown. On cut section was hemorrhagic. Ovary noted measuring 5 x 2.5 x 2.2 cm; cut section is solid-cystic. The section microscopically shows a fallopian tube with a wall showing hemorrhagic areas with intraluminal chorionic villi (Figure 6). Section from ovary was histologically unremarkable. The final assessment supported a left tubal ectopic pregnancy. In this case, the woman was G2P1L1 and had an early ectopic pregnancy at two different sites. The first ectopic pregnancy site was a scar; the second was in the left fallopian tube, a scarce instance with contemporaneous tubal and cesarean scar ectopic pregnancies. Successful patient management led to a positive outcome. After surgery, the patient's condition improved, and she was discharged.

## Discussion

An ectopic pregnancy occurs when a fertilized egg grows outside of the uterus. About 2% of pregnancies result in an ectopic pregnancy. Six percent of all pregnancy-related deaths are caused by ectopic pregnancies. Ninety-five percent of ectopic pregnancies are implanted in various fallopian tube segments, while the remaining 5% are deposited in the ovary, peritoneum, or a prior cesarean scar [2].

Ectopic pregnancies with a Caesarean scar are the most unusual type and in 1978, Larsen and Solomon were the first to report this case [5], and Seow et al. estimated its incidence to be 6.1% [6]. When a patient with many cesarean deliveries' history develops bleeding during the first trimester, this typically raises serious clinical concerns about ectopic pregnancy in the cesarean scar. Cesarean deliveries have increased cesarean scar ectopic pregnancy, which is still uncommon [2]. But in our case, ectopic pregnancy was present at two different sites: cesarean scar ectopic and left ectopic tubal pregnancy, which is a rare case.

The etiology of this condition is unknown. Scar implantation is probably caused by microtubular tract anomalies in the scar following past trauma from dilatation and curettage, cesarean section, myomectomy, hysterotomy, manual placenta removal, and aberrant placentation [7]. Ectopic scar pregnancies fall into two different kinds. After implantation in the previous scar, Type I spreads to the uterine cavity or the cervico-isthmic region (in cases of previous cesarean sections). Deep implantation of a gestational sac into an existing surgical scar defect following cesarean delivery, infiltrating growth into the uterine myometrium, and bulging from the uterine serosal surface are the causes of type II (CSP-II), which can lead to uterine rupture [8].

Symptoms are non-pathognomonic. This could result in uterine rupture, disseminated intravascular coagulation, life-threatening bleeding during pregnancy or curettage, and even death. Sometimes, an ectopic pregnancy will present with hemoperitoneum, shock, and excessive bleeding [9]. Hence, for successful therapy to prevent these adverse effects, early and accurate diagnosis is crucial [8]. The signs of a scar ectopic pregnancy include the following: (i) the myometrium of the lower uterine section had a gestational sac wedged eccentrically; (ii) an implant placed over a scar from a previous cesarean delivery; (iii) the uterine cavity is empty; and (iv) a previous cesarean scar site displays substantial blood flow on a color Doppler scan [2].

hCG levels, gestational age, and the presence of heart activity are considered while developing management strategies. The use of either medicinal or surgical intervention for treatment is possible. Treatment possibilities include selective uterine artery embolization with curettage or by methotrexate injections, bilateral hypogastric artery ligation, excision of trophoblastic tissues with dilatation, and evacuation under laparoscopic guidance. Pregnant women who are hemodynamically stable, have an unruptured cesarean scar, are less than eight weeks along in their pregnancy, and have a sonographic test that reveals the thickness of myometrium less than 2 mm between the CSP, and the bladder have been advised to receive conservative medical treatment. Surgery is suggested when medical treatment is ineffective, or a patient has hemodynamic instability [1].

This instance emphasizes the value of early detection and treatment of ectopic pregnancy in cesarean scars. U.S.G. was the first technique frequently used to identify ectopic scar pregnancies. According to reports, a sensitivity of 84.6% can be achieved for accurate early identification of these pregnancies using enhanced ultrasound imaging [10]. Sociologists with experience may provide an accurate diagnosis for prompt and efficient management. The diagnosis is made by transvaginal sonography, serum bhCG levels, clinical manifestations, history of scarring, and symptoms. Early in pregnancy, diagnosing an ectopic scar pregnancy is extremely simple. When a diagnosis may be made based on transvaginal color, magnetic resonance imaging (MRI) is advised. Doppler U.S.G. is challenging [8].

## Conclusions

Ectopic pregnancies present a diagnostic challenge that calls for radiologists and clinicians to maintain a high index of suspicion during imaging and follow-up managing women with associated risk factors. A missed diagnosis with delayed management may lead to massive hemorrhage, uterine rupture, and maternal death. Even in areas with limited resources, transvaginal scanning tools and training should be easily accessible. A screening tool for evaluating at-risk patients and a protocol for escalating to MRI for equivocal cases should be available at the point of care. Doctors must properly counsel the women about the many treatment options available based on their case reports that are currently available since there are no evidence-based recommendations available.
